# Nicotine promotes the development of oral leukoplakia via regulating peroxiredoxin 1 and its binding proteins

**DOI:** 10.1590/1414-431X2020e10931

**Published:** 2021-05-31

**Authors:** Moci Qi, Lingyu Li, Xiaofei Tang, Yunping Lu, Min Wang, Jing Yang, Min Zhang

**Affiliations:** 1 Beijing Institute of Dental Research, Beijing Key Laboratory, Beijing Stomatological Hospital and School of Stomatology, Capital Medical University, Dongcheng District, Beijing, China; 2 Department of Clinical Laboratory Medicine, Beijing Shijitan Hospital, Capital Medical University, Haidian District, Beijing, China

**Keywords:** Peroxiredoxin 1, Oral leukoplakia, Nicotine, Binding protein, Cell proliferation

## Abstract

Tobacco can induce reactive oxygen species (ROS) production extensively in cells, which is a major risk factor for oral leukoplakia (OLK) development. Peroxiredoxin 1 (Prx1) is a key antioxidant protein, upregulated in a variety of malignant tumors. We previously found that nicotine, the main ingredient of tobacco, promotes oral carcinogenesis via regulating Prx1. The aim of the present study was to screen and identify the Prx1 interacting proteins and investigate the mechanisms of nicotine on the development of OLK. Through liquid chromatography-tandem mass spectrometry combined with bioinformatics analysis, the candidate Prx1 interacting proteins of cofilin-1 (CFL1), tropomyosin alpha-3 chain (TPM3), and serine/threonine-protein phosphatase 2A 65 kDa regulatory subunit A alpha isoform (PPP2R1A) were screened in human dysplastic oral keratinocyte cells treated with nicotine. CFL1, TPM3, and PPP2R1A were highly expressed in human OLK tissues. The expression of CFL1 increased and the expression of PPP2R1A decreased in OLK of smokers compared to that in OLK of non-smokers. Nicotine upregulated CFL1 and downregulated PPP2R1A in 4-nitro-quinoline-1-oxide (4NQO)-induced OLK tissues in mice in part dependent on Prx1. Furthermore, the *in-situ* interaction of CFL1, TPM3, and PPP2R1A with Prx1 were validated in human OLK tissues. Our results suggested that tobacco might promote the development of OLK via regulating Prx1 and its interacting proteins CFL1 and PPP2R1A.

## Introduction

Tobacco is the major risk factor associated with oral leukoplakia (OLK) malignant transformation, which has an effect of promoting generation of reactive oxygen species (ROS) and causing oxidative damage to the oral mucosa (1,2). As the main addictive component of tobacco, nicotine affects tumorigenesis by regulation of cell survival, apoptosis, proliferation, metastasis, angiogenesis, and immunosurveillance (3). Peroxiredoxin 1 (Prx1) is an extensively distributed cellular antioxidant, which plays an important role in maintaining the intracellular redox homeostasis. Prx1 can remove excess ROS or act as a molecular chaperone delivering peroxide signals when ROS level is abnormally elevated (4,5). Its high abundance in various tumor tissues suggests that Prx1 is closely related with the development of tumors (6). In a previous study, we found that Prx1 upregulated significantly in OLK and oral squamous cell carcinoma (OSCC) tissues and the expression of Prx1 in OLK and OSCC tissues of smokers was significantly higher than that in non-smokers. Additionally, nicotine upregulated the expression of Prx1 in oral precancerous lesion dysplastic oral keratinocyte (DOK) and OSCC cells and promoted the development of oral precancerous lesions by regulating Prx1 expression involved in cell proliferation and apoptosis (7,8). These results suggest that tobacco might promote the development of OLK by regulating Prx1. However, extensive research is necessary to elucidate the molecular function of Prx1 in tobacco-related OLK.

In the present study, we screened and validated the Prx1 binding proteins of cofilin-1 (CFL1), tropomyosin alpha-3 chain (TPM3), and serine/threonine-protein phosphatase 2A 65 kDa regulatory subunit A alpha isoform (PPP2R1A), and examined the protein expression in both OLK tissues in the Prx1^+/+^ and Prx1^+/−^ mouse model and human OLK tissues to further investigate the underlying mechanisms for tobacco promoting the development of OLK by regulating Prx1. Our study revealed some new molecular mechanisms of OLK pathogenesis.

## Material and Methods

### Cell culture and treatment

Human DOK cells (provided by Professor Xiaoxin Chen from North Carolina Central University, USA) were maintained in high-glucose Dulbecco's modified Eagle's medium (DMEM) supplemented with 10% (v/v) fetal bovine serum (FBS) (Gibco, USA), containing 100 units/mL penicillin and 100 µg/mL streptomycin. DOK cells were treated with 1 μM/L of nicotine (Sigma-Aldrich, USA) for 7 days in a 5% CO_2_ atmosphere at 37°C.

### LC-MS/MS and bioinformatics analysis

The total protein of the nicotine treatment group and the control group was extracted and incubated with anti-Prx1 antibody (1:1000, Abcam, USA) for Co-IP assay to enrich Prx1 interacting proteins. The peptides were analyzed by liquid chromatography-tandem mass spectrometry (LC-MS/MS), and Mascot (http://www.matrixscience.com/) and UniProt (https://www.uniprot.org/) databases were used to identify proteins from the peptide sequences. The proteins with unique peptide counts ≥1 were considered receivable. Gene ontology (GO), Kyoto Encyclopedia of Genes and Genomes (KEGG) pathway, and protein-protein interaction (PPI) analysis were performed. Prx1 interacting proteins related to cell proliferation were screened based on bioinformatics analysis results.

### Animal assay

An animal model of 4-nitro-quinoline-1-oxide (4NQO)-induced OLK established in the tongue tissues of wild-type (Prx1^+/+^) and Prx1 knockdown (Prx1^+/−^) mice (9) was used in the study. Wild type C57BL/6 mice were purchased from Vital River Laboratory Animal Technology (China). One hundred and forty 6-8-week-old C57BL/6 mice (250-300 g) were maintained under standard conditions in accordance with institutional guidelines. The experimental protocol was approved by the Ethics Committee of Capital Medical University (approval No. KQYY-201503-010, protocol # NSFC 81470752).

Both Prx1^+/+^ and Prx1^+/−^ mice were randomly divided into four groups: control (n=10), treated with nicotine (n=20), treated with 4NQO (n=20), and treated with 4NQO+nicotine (n=20). The control group received vehicle (distilled water) treatment. 4NQO group was treated with 4NQO solution (50 µg/mL) in drinking water. The nicotine group received 5% nicotine treatment on the tongue mucosa 3 times a week. The 4NQO+nicotine group received both 4NQO and nicotine treatment as above. At the end of treatment (16 weeks), the tongues of all mice were removed after the mice were sacrificed. Histological observation and pathological diagnosis results showed that the oral precancerous lesions were induced successfully by 4NQO on the tongue mucosa of Prx1^+/+^ and Prx1^+/−^ mice.

### Patients and tissue specimens

Twenty-five OLK and ten normal oral mucosa samples were obtained from patients who underwent treatment at Beijing Stomatological Hospital, Capital Medical University during 2015-2017. The OLK specimens in the smoking group were from 10 men ranging in age from 54 to 77 years old, and specimens in the non-smoking group were from 7 men and 8 women ranging in age from 49 to 89 years old. The normal oral mucosa from 6 women and 4 men (age range 24-54 years old) were used as control. The present study was approved by the Human Research Ethics Committee of Capital Medical University School of Stomatology and all subjects signed informed consents.

### HE and immunohistochemical staining

Pathological diagnosis on tissue lesions was made based on HE staining according to the World Health Organization classification of head and neck tumors (4th Edition, 2017) (9). The histological changes in tongue mucosa and the degree of lesion were recorded as normal mucosa, hyperplasia, dysplasia, and OSCC. Immunohistochemistry (IHC) streptavidin-peroxidase two-step staining was performed to detect the expression of Prx1 binding proteins. After incubation in 3% hydrogen peroxide at room temperature for 15 min, the sections were incubated with primary antibodies to PPP2R1A (1:400), TPM3 (1:50), or CFL1 (1:400) (all from Abcam) overnight at 4°C. Following incubation in secondary anti-rabbit IgG (Abcam) for 30 min, slides were stained with chromogen diaminobenzidine (DAB) (Maixin, China) staining and hematoxylin redying. Three representative regions were selected at 200× magnification under the microscope Olympus BX61 (Olympus, Japan). Image Pro Plus software (Media Cybernetics, USA) was used for quantitative analyses and MOD (integral optical density (IOD)/measurement area) was calculated.

### Duolink *in situ* interaction assay in patient tissue specimens

A pair of proximity ligation assay (PLA) probes targeting Prx1 and its interacting proteins were used in this assay. When the two PLA probes are in close proximity to each other, the oligonucleotide in PLA probes form a DNA circle template for the amplification with fluorescent signals. After being dewaxed and dehydrated with xylene and ethanol, tissues were permeabilized in PBS for 20 min at room temperature. Two probes (mouse- or rabbit-derived) with oligonucleotide-labeled secondary antibodies were added after incubation with primary antibodies against Prx1 (1:1000), CFL1 (1:200), TPM3 (1:200), and PPP2R1A (1:200). Blocking, staining, hybridization, ligation, amplification, and detection were performed in a humidity chamber. Fluorescence was visualized in the microscope Olympus BX61.

### Statistical analysis

Differences were analyzed by one-way analysis of variance (SPSS v17.0, IBM, USA), and a P-value less than 0.05 was considered statistically significant.

## Results

### Screening of Prx1 interacting proteins related to cell proliferation

As analyzed by the Mascot database and UniProt database, 358 and 580 of candidate Prx1 interacting proteins were identified in the control and nicotine treatment groups, respectively. Through bioinformatics analysis, CFL1, TPM3, and PPP2R1A were screened out from the candidate Prx1 interacting proteins, which were related to cell proliferation (Tables 1 and 2).

**Table 1:** Spectrum identification of peroxiredoxin 1 (Prx1) interacting proteins.

Accession	Description	Gene name	MW [kDa]	PI	Coverage	Unique peptides
P23528	Cofilin-1	CFL1	18.5	8.09	43.98	6
P06753	Tropomyosin alpha-3 chain	TPM3	32.9	4.72	22.81	1
P30153	Serine/threonine-protein phosphatase 2A 65 kDa regulatory subunit A alpha isoform	PPP2R1A	65.3	5.11	3.90	2

MW: molecular weight; PI: isoelectric point.

**Table 2:** Gene ontology (GO) analysis of peroxiredoxin 1 (Prx1) interacting proteins.

Gene name	GO number	GO term
CFL1	GO:0043066	Negative regulation of apoptotic process
	GO:0022604	Regulation of cell morphogenesis
	GO:0030010	Establishment of cell polarity
TPM3	GO:0006928	Cellular component movement
	GO:0006915	Apoptotic process
PPP2R1A	GO:0030308	Negative regulation of cell growth
	GO:0030155	Regulation of cell adhesion
	GO:0040008	Regulation of growth
	GO:0000188	Inactivation of MAPK activity

### Effect of nicotine on expression of CFL1, PPP2R1A, and TPM3 in induced OLK in Prx1^+/+^ mice

**Figure 1:**
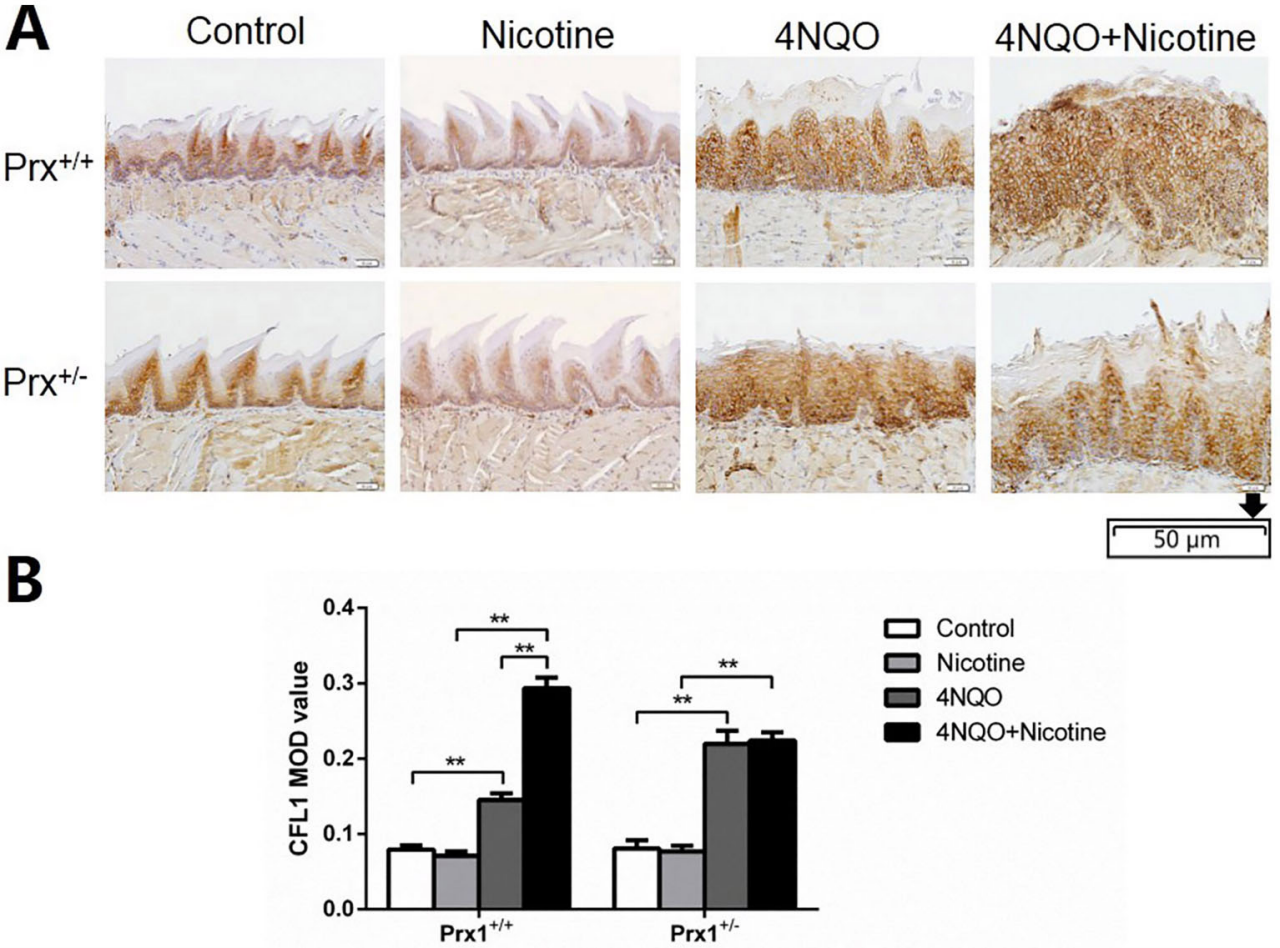
Immunohistochemical analysis of CFL1 in the tongue oral leukoplakia tissues of Prx1^+/+^ and Prx1^+/-^ mice. **A**, Representative immunohistochemistry images of CFL1 (×200, scale bar 50 μm). **B**, The integral optical density (IOD)/measurement area (MOD) values are reported as means±SE. **P<0.01 (ANOVA). 4NQO: 4-nitro-quinoline-1-oxide.

Histological observation and pathological diagnosis showed that all animals treated with either 4NQO or 4NQO+nicotine developed OLK with epithelial hyperplastic and dysplastic lesions in the tongue mucosa. As shown in Figure 1, we found that mice treated with 4NQO showed significantly higher expression of CFL1 than control animals (P<0.01) both in Prx1^+/+^ and Prx1^+/-^ mice. Additionally, 4NQO+nicotine treatment promoted further expression of CFL1 compared to treatment with 4NQO alone in Prx1^+/+^ mice.

**Figure 2:**
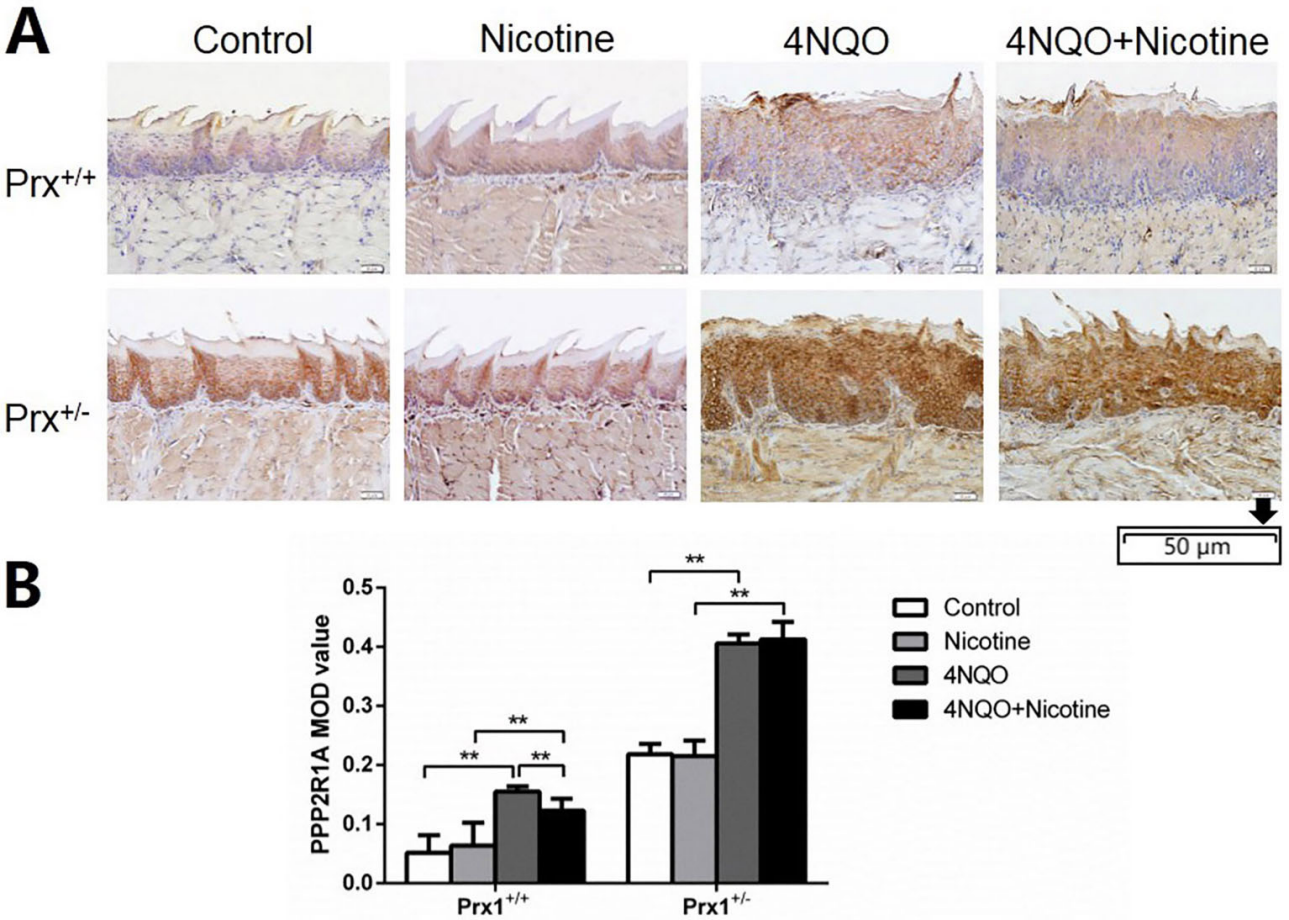
Immunohistochemical analysis of PPP2R1A in the tongue oral leukoplakia tissues of Prx1^+/+^ and Prx1^+/−^ mice. **A**, Representative immunohistochemistry images of PPP2R1A (× 200, scale bar 50 μm). **B**, The integral optical density (IOD)/measurement area (MOD) values are reported as means±SE. **P<0.01 (ANOVA). 4NQO: 4-nitro-quinoline-1-oxide.

As shown in Figure 2, compared with the control, animals treated with 4NQO expressed a significantly higher level of PPP2R1A both in tongue tissues of Prx1^+/+^ and Prx1^+/-^ mice (P<0.01). However, in Prx1^+/+^ mice, the PPP2R1A expression was significantly reduced by treating with 4NQO+nicotine compared to treating with 4NQO alone.

**Figure 3:**
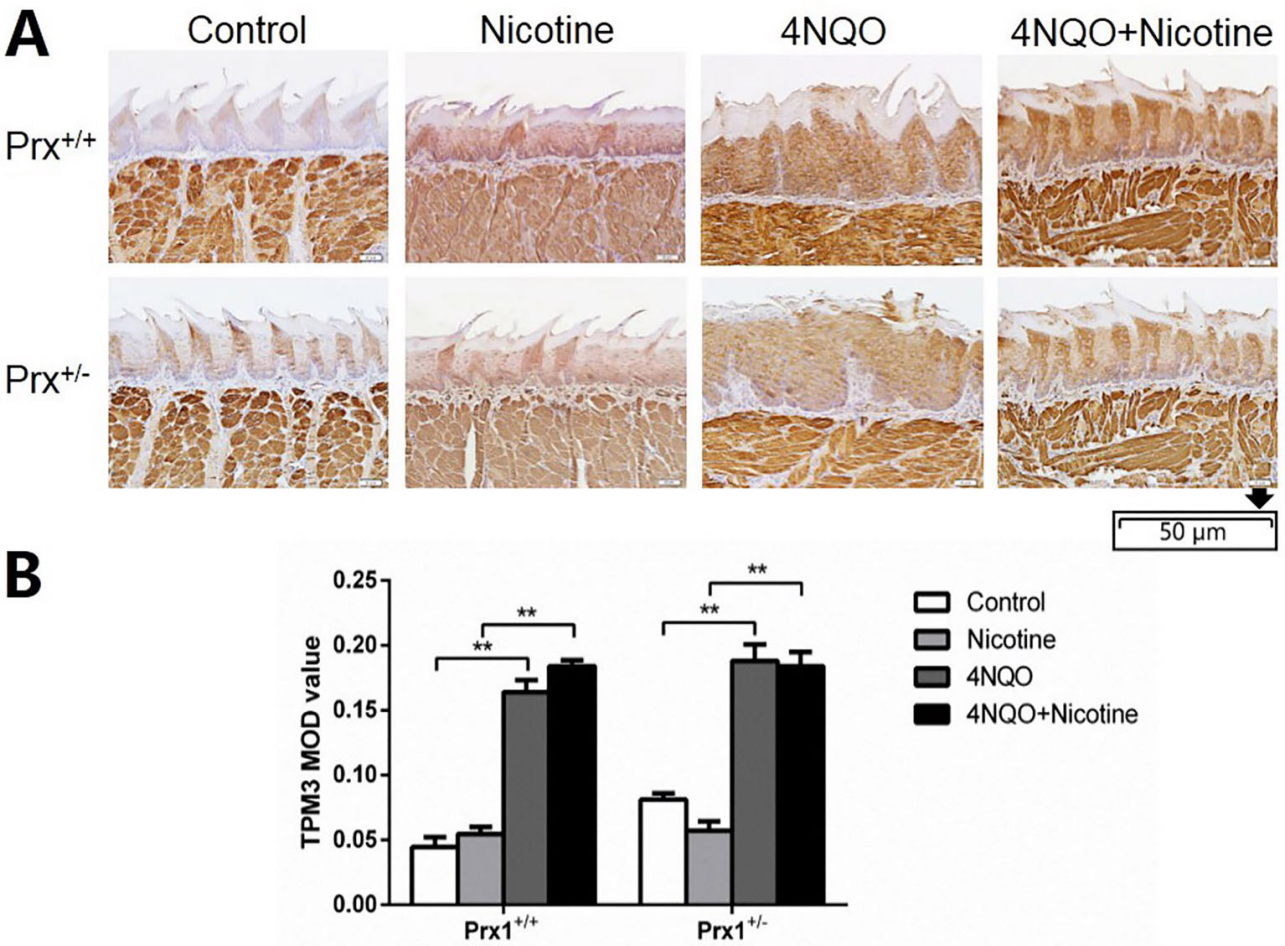
Immunohistochemical analysis of TPM3 in the tongue oral leukoplakia tissues of Prx1^+/+^ and Prx1^+/-^ mice. **A**, Representative images of TPM3 (×200, scale bar 50 μm). **B**, The integral optical density (IOD)/measurement area (MOD) values are reported as means±SE. **P<0.01 (ANOVA). 4NQO: 4-nitro-quinoline-1-oxide.

TPM3 was upregulated in 4NQO-induced OLK in both Prx1^+/+^ and Prx1^+/-^ mice (Figure 3, P<0.01), but no significant change in TPM3 expression was detected comparing tongue tissues treated with 4NQO+nicotine to those treated with 4NQO in Prx1^+/+^ mice.

### CLF1, PPP2R1A, and TMP3 expression in induced OLK in Prx1^+/-^ mice treated with nicotine

As shown in Figures 1, 2, and 3, no significant changes in the expression of CFL1, PPP2R1A, and TPM3 were found in 4NQO+nicotine treatment of Prx1^+/-^ mice compared to 4NQO group.

### Verification of the Prx1 interacting proteins in human OLK tissues

**Figure 4:**
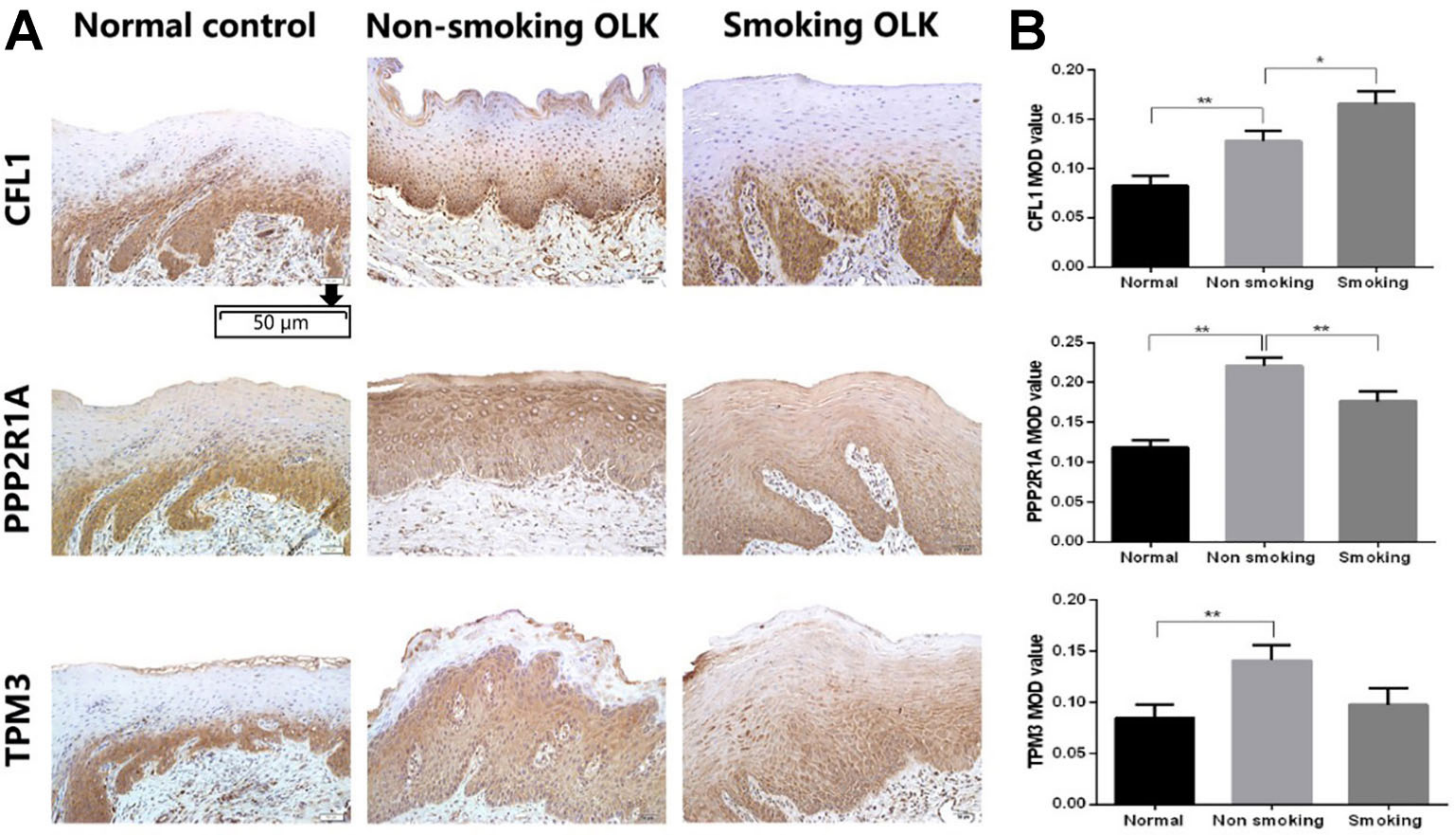
Immunohistochemical analysis of CFL1, PPP2R1, and TPM3 in human normal mucosa and oral leukoplakia (OLK) tissues of smokers and non-smokers. **A**, Representative images of PPP2R1, TPM3, and CFL1 in normal mucosa and OLK tissues (×200, scale bar 50 μm). **B**, The integral optical density (IOD)/measurement area (MOD) values are reported as means±SE. *P<0.05; **P<0.01 (ANOVA). 4NQO: 4-nitro-quinoline-1-oxide.

As shown in Figure 4, the expression of CFL1, PPP2R1A, and TPM3 significantly increased in OLK compared to normal tissues. When comparing OLK of smokers and non-smokers, the expression level of CFL1 was higher (P<0.05), while the expression of PPP2R1A was lower (P<0.01). However, no significant difference in TPM3 expression was detected.

**Figure 5:**
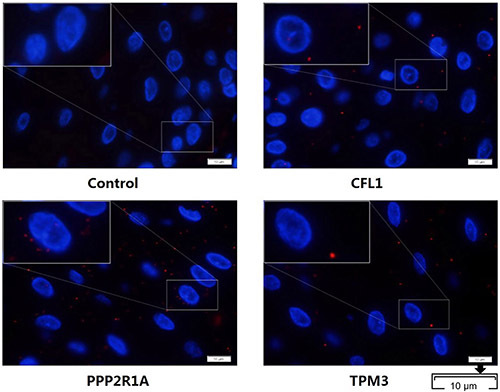
Duolink analysis. The interactions of PPP2R1A, TPM3, and CFL1 with Prx1 in human oral leukoplakia tissues were detected by *in situ* Duolink analysis. Proximity ligation assay signals are shown in red and the nuclei are stained in blue (×1000, scale bar 10 μm).

We then used an *in situ* Duolink assay to detect 3 candidate binding proteins in the above human OLK tissues of smokers. Positive red spots were found in these tissues, which confirmed that there are interactions of PPP2R1A, CFL1, and TPM3 with Prx1 in human OLK tissues (Figure 5). These results revealed a novel role of Prx1 involved in OLK development by interacting with CFL1, PPP2R1A, and TPM3.

## Discussion

Cell proliferation is a vital indicator for understanding the mechanisms of function of certain genes, proteins, and pathways involved in cell survival and death. The cytoskeleton may induce cell proliferation through modulating cell hardness, and mechanical forces in cells can influence the cytoskeleton assembly, which in turn affects cell proliferation. In this study, three Prx1 interacting proteins, CFL1, PPP2R1A, and TPM3, related to cell proliferation, were screened out by mass spectrometry followed with GO and KEGG pathway analysis. According to existing reports, CFL1, PPPR1A, and TPM3 are all regulatory proteins in the dynamics of actin filaments. To confirm the binding of CFL1, PPPR1A, and TPM3 to Prx1, Duolink assay was conducted in human OLK tissues. Duolink analysis is used to detect the interaction between two proteins with high sensitivity and specificity *in situ*. The results can be interpreted with countable spots, each representing a single-molecule event. Duolink technique provides the benefits of traditional Co-IP plus the information of localization and the ability to study transient, weak interactions. We observed the interaction of CFL1, PPPR1A, and TPM3 with Prx1, which indicated that Prx1 performs the important function through its interaction network in OLK development; the bindings of CFL1 and PPPR1A with Prx1 were especially noteworthy in tobacco-associated OLK.

Some preliminary studies of CFL1 and PPP2R1A involved in cell proliferation have been reported. In endometriotic patients, the phosphorylation of CFL1 regulates cell proliferation through the LIMK1/cofilin1 pathway (10). In eutopic endometrium of endometriosis patients, silencing CFL1 could block PDGF-induced proliferation (11). According to reports, expression of CFL1 varies in carcinomas including prostate, breast, lung, colorectal, and oral cancers, and the expression of CFL1 was increased in bladder cancer tissues compared with the precancerous lesion (12–14). Activated CFL1 participates in some essential biological processes of malignant tumors including proliferation, apoptosis, invasion, and chemo-resistance (15). PPP2R1A, an isoform of PP2A, is an important serine/threonine protein phosphatase and is considered to be a tumor suppressor in some human malignancies (16). The mutations in PPP2R1A have been identified in multiple cancers, but the effects of these mutations on PP2A function need to be fully elucidated. Mutations of PPP2R1A promote cells proliferation by upregulating the phosphorylation levels of Akt, ERK, and WNK1 in gastrointestinal stromal tumors and by interactions with the PP2A inhibitor TIPRL1 in uterine cancer (17,18). In alveolar rhabdomyosarcoma, silencing of PPP2R1A significantly increased cell growth, suggesting the tumor-suppressive function of PPP2R1A (19). Our results showed that the expression of CFL1 and PPP2R1A was higher in human and mouse OLK tissues than that in normal tissues, indicating their roles in the development of oral precancerous lesions. In addition, nicotine and Prx1 were associated with an upregulation of CFL1 and a downregulation of PPP2R1A in OLK. Notably, in Prx1^+/-^ mice, this association between nicotine and CFL1 or PPP2R1A was attenuated in OLK tissues, further highlighting the importance of the Prx1 interaction network in OLK. The results suggested that nicotine upregulated CFL1 and downregulated PPP2R1A both depending on Prx1 and thus promoted the development of OLK. CFL1 or PPP2R1A could be a marker for the treatment of tobacco-related OLK.

We also observed a higher expression of TPM3 in human and mouse OLK tissues in this study. TPM3 belongs to the tropomyosin super family, which can be viewed as a universal regulator of the actin cytoskeleton for cellular functions such as cell proliferation (20). The expression levels of TPM3 are higher in stage III esophageal squamous cell carcinoma tissue compared with stage I (21), and glucose glycated TPM3 suppresses colon cancer cell Caco-2 proliferation (22). In immortalized mouse embryonic fibroblasts, TPM3 is partly responsible for cell proliferation via regulating the interaction between pERK and Imp7, which is overridden by Ras transformation (23). Transfection of TPM3-anaplastic lymphoma kinase (ALK) fusion gene results in the loss of contact inhibition in NIH3T3 cells (24). The results of the current study suggested that TPM3 may be an important partner of the Prx1 network in regulating cell proliferation in OLK. However, nicotine had no significant effect on expression of TPM3 in 4NQO-induced OLK tissues. Further studies are needed to explore the mechanism of Prx1/TPM3 in tobacco-related OLK.

In this study, for the first time, we found that nicotine increased expression of CFL1 and decreased expression of PPP2R1A in 4NQO-induced OLK tissues by regulating Prx1 in mice. The binding of CFL1, PPP2R1A, and TPM3 to Prx1 was confirmed in human OLK tissues. It suggested that tobacco promoted the development of OLK via regulating Prx1 and its interacting proteins CFL1 and PPP2R1A. Results of this study may be helpful to develop strategies for OLK prevention targeting Prx1 and its interacting proteins.
